# Supplementary material for: Identifying Wild Versus Cultivated Gene-Alleles Conferring Seed Coat Color and Days to Flowering in Soybean
Source: Int J Mol Sci. 2021 Feb 4;22(4):1559. doi: 10.3390/ijms22041559 (PMC7913812; doi:10.3390/ijms22041559)
Supplement: Supplementary file 1 [file ijms-22-01559-s001.pdf]

Supplementary material

Supplemental Tables

Table S1 Comparisons of genome composition among the five CSSL populations
Table S2 Summary of the sequencing data of *SojaCSSLP5* along with its two parents
Table S3 Summary of SNPs identified in parents and *SojaCSSLP5*
Table S4 Descriptive statistics of the DTF in the three environments
Table S5 Joint analysis of variance of days to flowering in *SojaCSSLP5*
Table S6 The expression of genes associated with seed coat color and days to flowering
Table S7 The allelic variations of candidate genes between *N24852* and *NN1138-2*

Table S1 Comparisons of genome composition among the five CSSL populations

Population	No. of CSSLs	NN1138-2 (%)	N24852 (%)	Heterozygous segment (%)	Reported by
SojaCSSLP1	151	95.09	3.68	1.18	Wang et al. (2013)
SojaCSSLP2	152	94.66	4.02	0.96	Xiang et al. (2015)
SojaCSSLP3	158	95.04	3.73	1.19	He et al. (2014)
SojaCSSLP4	195	95.33	3.67	0.98	Yang et al. (2017)
SojaCSSLP5	177	95.01	4.97	0.02	Present study

Table S2 Summary of the sequencing data of *SojaCSSLP5* along with its two parents

Material	Clean reads	Mapping reads	Mapping rate (%)	GC content (%)	Average depth
NN1138-2	80,336,554	79,043,720	98.39	38.61	11.39
N24852	70,508,508	69,128,829	98.04	35.57	9.51
SojaCSSLP5 (average)	20,122,918	19,799,715	98.39	30.03	3.04

Clean reads: the number of high quality bases after filtering the raw data; Mapping reads: the number of reads that mapped to the Williams 82 reference genome; Mapping rate: the ratio of Mapping reads to the total clean reads; Average depth: the number of times of a base was sequenced.

Table S3 Summary of SNPs identified in parents and *SojaCSSLP5*

**SNP categories**	**Fields**	**NO. SNP**	
		Parents	CSSLs
Exon	Nonsynonymous variant	59709	53539
	Synonymous variant	44547	41663
	Start lost	141	123
	Stop gained	1432	1280
	Stop lost	268	240
	Stop retained variant	65	61
	Initiator codon variant	24	22
Splice site	Splice region variant	7510	6891
	Splice donor variant	414	383
	Splice acceptor variant	444	386
Intron	Intron variant	158054	132415
UTR	5 prime UTR variant	23676	21065
	5 prime UTR premature start codon gain variant	3913	3503
	3 prime UTR variant	42464	38005
Intergenic	Upstream gene variant	982404	693138
	Downstream gene variant	520633	392082
	Intergenic region	**1498379**	**1182630**
Total		3344077	2567426

UTR, untranslated region. Upstream or downstream is within a 2-kb region upstream or downstream of the transcription start site.

Table S4 Descriptive statistics of the DTF in the three environments

ENV	Parent (day)		*SojaCSSLP5*			
	NN1138-2	*N24852*	Range (day)	Mean(day)	CV(%)	h^2(%)
2016JP	54.0	68.0	51.0-63.0	54.1	5.3	92.0
2017DT	55.0	64.7	50.0-62.0	54.1	4.9	91.2
2018DT	52.0	68.0	50.0-64.0	52.8	6.1	98.7
Whole	53.7	66.9	50.0-64.0	53.7	5.4	95.6

2016JP, 2017DT and 2018DT, represent the environments of 2016 Jiangpu, 2017 Dangtu and 2018 Dangtu, respectively. CV represents coefficient of variation. h^2 represents heritability.

Table S5 Joint analysis of variance of days to flowering in *SojaCSSLP5*

Source	DF	SS	MS	*F* value	*P* value
Env	2	608.56	304.28	249.53	< .0001
Rep (Env)	6	106.32	17.72	14.53	< .0001
CSSL	176	11208.72	63.69	52.23	< .0001
Env × CSSL	352	947.44	2.69	2.21	< .0001
Error	1056	1287.68	1.22		

F-tests were carried out using the GLM procedure under Model III ANOVA. Env, environment. Rep (Env) means replications within Env. CSSL, chromosome segment substitution line. DF, degree of freedom. SS, sum of squares. MS, mean of squares.

Table S6 The expression of genes associated with seed coat color and days to flowering

Locus	Gene	Parents	Tissue (FPKM)							
			Leaf	Flower	14seed	21seed	28seed	35seed	7pod	21pod
Gm01_LDB_74	Glyma.01G194500	N24852	4.00	11.31	6.48	1.51	0.36	0.00	9.40	5.44
		NN1138-2	11.39	10.38	7.39	4.42	0.73	0.79	10.12	4.96
	Glyma.01G194600	N24852	7.52	6.62	9.26	5.41	5.21	3.12	10.87	12.54
		NN1138-2	6.80	7.54	13.11	10.78	3.13	4.81	9.26	11.60
	Glyma.01G194700	N24852	7.18	1.88	7.73	7.71	4.89	4.26	6.83	4.25
		NN1138-2	5.24	4.88	3.15	13.36	5.96	4.94	5.67	3.69
	Glyma.01G194800	N24852	3.21	2.21	2.67	1.19	2.95	1.13	4.43	1.95
		NN1138-2	2.38	2.79	8.48	4.22	0.39	1.67	5.43	3.04
	Glyma.01G194900	N24852	6.74	46.82	5.94	7.44	10.38	6.14	4.39	10.00
		NN1138-2	4.79	28.71	5.06	5.52	2.43	7.99	4.69	5.41
	Glyma.01G195000	N24852	0.16	2.28	8.65	6.62	2.25	1.08	11.31	1.40
		NN1138-2	6.37	5.57	5.87	11.13	2.70	1.59	8.48	5.27
	Glyma.01G195100	N24852	0.15	0.21	0.68	0.21	0.64	0.22	1.38	0.49
		NN1138-2	1.44	0.60	2.47	0.95	0.39	0.32	1.60	0.27
	Glyma.01G195200	N24852	0.50	2.47	1.76	0.56	0.66	0.32	3.03	0.32
		NN1138-2	0.44	2.89	1.72	1.21	0.24	0.20	2.88	1.48
	Glyma.01G195400	N24852	9.12	34.91	14.78	6.72	10.26	24.22	13.74	28.74
		NN1138-2	8.93	39.93	12.71	10.40	8.14	6.54	11.89	25.04
	Glyma.01G195500	N24852	0.05	0.25	0.19	0.07	0.30	0.54	0.05	0.10
		NN1138-2	0.05	0.25	0.10	0.32	0.04	0.07	0.03	0.05
	Glyma.01G195600	N24852	4.85	0.76	0.39	0.98	0.61	0.37	0.05	0.65
		NN1138-2	0.83	0.54	0.09	0.19	0.58	1.06	0.11	0.38
	Glyma.01G195700	N24852	0.14	0.14	0.49	1.03	0.00	0.17	0.29	0.07
		NN1138-2	0.09	0.23	0.20	0.20	0.07	0.00	0.17	0.09
	Glyma.01G195900	N24852	0.04	0.82	0.34	0.43	0.09	0.00	2.61	0.05
		NN1138-2	0.28	1.28	2.35	0.78	0.25	0.08	6.04	0.81
	Glyma.01G196000	N24852	3.35	5.10	3.88	2.03	11.85	7.15	18.03	2.61
		NN1138-2	1.93	6.14	8.11	3.57	0.59	2.09	11.12	9.40
	Glyma.01G196300	N24852	7.30	7.41	12.79	6.24	7.81	3.34	26.73	18.16
		NN1138-2	15.76	10.36	23.38	13.00	2.30	6.19	23.83	12.70
	Glyma.01G196600	N24852	18.45	9.86	22.75	6.88	3.67	3.34	6.13	10.90
		NN1138-2	20.82	18.13	32.28	20.90	4.63	5.08	12.01	43.96
	Glyma.01G196800	N24852	0.61	11.68	4.19	0.00	0.00	0.00	3.72	0.36
		NN1138-2	12.14	8.13	0.38	0.07	0.00	0.00	2.99	9.73
	Glyma.01G197100	N24852	7.26	7.68	5.54	2.45	7.39	20.43	6.82	10.93
		NN1138-2	5.92	8.25	6.41	5.34	2.33	3.82	7.73	8.02
	Glyma.01G197200	N24852	0.05	0.48	0.05	0.07	1.22	9.75	0.05	0.09
		NN1138-2	0.00	0.64	0.26	0.04	0.00	0.04	0.11	0.17
	Glyma.01G197300	N24852	1.40	1.16	0.64	0.66	1.29	2.78	0.19	0.16
		NN1138-2	0.69	1.36	0.61	1.03	1.46	1.47	0.19	0.26
	Glyma.01G197500	N24852	1.64	6.18	11.40	12.52	4.06	0.06	40.01	2.25
		NN1138-2	19.20	21.56	21.19	3.97	3.82	0.56	21.39	28.32
	Glyma.01G197600	N24852	2.45	4.30	4.42	1.92	0.85	0.01	5.56	1.18
		NN1138-2	1.36	5.53	5.57	4.77	0.91	0.93	3.94	3.64
	Glyma.01G197700	N24852	0.10	0.12	0.44	0.19	0.11	0.15	0.08	0.34
		NN1138-2	0.03	0.14	0.05	0.05	0.05	0.04	0.03	0.66
	Glyma.01G197800	N24852	3.03	9.27	27.92	11.71	9.87	1.43	29.68	7.90
		NN1138-2	8.88	9.08	40.12	38.08	5.99	10.12	21.44	11.70
	Glyma.01G198100	N24852	4.63	4.18	0.95	0.47	0.30	0.00	12.57	0.22
		NN1138-2	23.95	4.42	1.95	2.17	0.28	0.88	20.16	0.12
	Glyma.01G198200	N24852	0.00	0.05	0.06	0.04	0.09	0.06	0.03	0.08
		NN1138-2	0.23	1.30	12.92	0.78	0.99	1.29	1.95	0.30
	Glyma.01G198400	N24852	0.00	0.00	0.00	0.00	0.28	0.00	0.27	0.00
		NN1138-2	0.64	2.28	7.71	0.48	0.25	0.92	1.54	1.28
	Glyma.01G198500	N24852	113.96	8.69	14.96	8.76	7.05	2.57	16.18	11.98
		NN1138-2	109.98	20.01	3.55	9.02	4.25	9.49	24.26	32.42
	Glyma.01G198600	N24852	1.68	1.15	1.37	0.98	6.78	65.23	1.58	2.08
		NN1138-2	1.46	1.69	2.51	1.39	0.35	1.50	2.81	4.76
	Glyma.01G198900	N24852	0.65	3.74	3.55	0.71	0.69	0.68	6.24	2.61
		NN1138-2	0.49	2.23	6.28	4.07	0.59	1.26	8.23	2.84
	Glyma.01G199000	N24852	1.23	0.71	1.08	0.66	0.81	0.39	1.24	1.63
		NN1138-2	0.90	1.68	2.27	1.62	0.88	1.45	2.62	2.72
	Glyma.01G199100	N24852	12.51	12.13	16.49	8.99	7.72	3.38	21.41	20.40
		NN1138-2	14.30	15.11	26.28	15.84	6.23	8.59	21.08	12.54
	Glyma.01G199200	N24852	6.59	0.49	1.34	0.20	0.17	0.00	0.91	0.54
		NN1138-2	5.04	0.95	4.44	1.48	0.26	0.64	1.13	1.71

Locus	Gene	Parents	Tissue (FPKM)							
			Leaf	Flower	14seed	21seed	28seed	35seed	7pod	21pod
	Glyma.01G199300	N24852	0.62	0.17	0.27	0.83	0.49	0.28	1.10	0.24
		NN1138-2	1.33	0.83	2.63	0.62	0.36	0.62	1.37	0.60
	Glyma.01G199400	N24852	26.78	1.45	1.32	0.00	0.00	0.02	0.04	2.76
		NN1138-2	12.52	3.16	0.00	0.00	0.04	0.00	0.02	4.29
	Glyma.01G199600	N24852	0.47	1.46	4.76	3.29	1.80	0.19	4.99	1.98
		NN1138-2	0.45	1.73	13.35	6.26	2.91	1.27	5.39	2.38
	Glyma.01G199700	N24852	71.63	8.21	21.84	5.22	0.45	0.20	47.03	22.64
		NN1138-2	280.92	8.98	1.81	15.38	3.75	0.86	35.94	12.86
	Glyma.01G199900	N24852	4.55	8.55	6.76	4.91	4.87	0.97	3.89	4.19
		NN1138-2	3.99	8.51	4.87	6.01	2.52	3.84	4.79	4.44
	Glyma.01G200000	N24852	9.68	7.78	8.94	5.47	3.52	2.22	10.91	9.28
		NN1138-2	13.31	8.50	12.68	11.91	6.25	3.05	12.09	10.99
	Glyma.01G200100	N24852	9.72	0.86	0.93	9.77	0.38	0.23	0.02	0.68
		NN1138-2	0.79	2.15	0.73	0.47	2.71	2.29	0.07	4.97
	Glyma.01G200200	N24852	24.11	1.94	4.04	1.31	0.60	0.14	3.06	10.84
		NN1138-2	8.90	3.38	1.85	0.80	0.28	0.64	3.06	9.15
	Glyma.01G200500	N24852	2.48	8.53	7.94	0.79	2.18	1.07	3.55	19.70
		NN1138-2	4.50	6.58	2.49	2.98	0.74	1.93	4.86	17.57
	Glyma.01G200600	N24852	0.00	0.00	1.08	0.00	0.00	0.00	0.00	0.00
		NN1138-2	0.00	0.00	16.02	0.22	0.00	0.00	0.00	0.00
	Glyma.01G200700	N24852	7.07	17.70	14.85	10.73	8.70	3.71	14.81	13.80
		NN1138-2	9.61	13.03	27.56	18.18	5.67	7.33	13.02	10.09
	Glyma.01G200800	N24852	8.38	8.51	30.78	5.55	3.84	0.02	27.12	5.18
		NN1138-2	14.82	13.01	18.78	28.35	4.28	5.91	29.87	26.16
	Glyma.01G200900	N24852	1.95	1.34	2.82	1.93	2.25	3.19	3.32	3.82
		NN1138-2	1.35	1.80	4.12	2.94	1.33	1.50	3.65	2.58
	Glyma.01G201000	N24852	91.95	12.36	41.82	13.40	36.08	51.38	64.77	26.66
		NN1138-2	319.99	28.06	45.01	67.34	7.58	11.64	76.17	37.98
	Glyma.01G201100	N24852	8.49	14.96	9.47	2.74	5.75	6.27	8.67	12.57
		NN1138-2	7.52	11.65	6.25	7.25	2.40	5.95	8.39	12.87
	Glyma.01G201200	N24852	19.66	18.58	24.78	24.99	35.19	48.58	36.37	30.93
		NN1138-2	32.48	22.74	28.61	24.31	11.81	18.57	36.90	22.51
	Glyma.01G201300	N24852	1.58	1.38	3.54	1.27	2.04	1.09	4.30	3.08
		NN1138-2	1.07	1.45	4.75	4.54	0.93	2.02	3.57	3.32
	Glyma.01G201400	N24852	3.22	2.24	8.65	3.85	2.97	2.06	7.37	6.36
		NN1138-2	3.67	3.99	9.61	8.28	3.52	4.99	8.40	6.85
	Glyma.01G202000	N24852	10.48	7.57	9.12	2.18	3.49	1.05	5.14	6.90
		NN1138-2	3.42	4.78	2.11	4.80	0.78	3.10	4.48	4.79
Gm08_LDB_32	Glyma.08G109200	N24852	3.88	1.97	0.93	9.44	7.10	0.25	3.43	0.47
		NN1138-2	3.01	2.92	1.46	10.71	3.39	2.50	0.31	2.35
	Glyma.08G109300	N24852	1.38	1.35	1.48	13.14	2.01	0.04	0.86	0.14
		NN1138-2	2.98	2.34	1.47	6.61	7.55	2.47	0.33	0.58
	Glyma.08G109400	N24852	1.52	1.68	1.80	19.09	1.60	0.11	0.26	0.03
		NN1138-2	6.78	5.09	9.45	39.32	6.51	4.81	0.35	0.73
	Glyma.08G109500	N24852	0.36	0.79	0.35	1.74	1.64	0.07	0.13	0.06
		NN1138-2	2.29	1.70	0.28	1.52	0.38	0.48	0.00	0.09
	Glyma.08G110300	N24852	0.14	0.09	0.08	0.70	0.42	0.00	0.12	0.03
		NN1138-2	0.10	0.26	0.24	0.48	0.85	0.23	0.05	0.05
	Glyma.08G110400	N24852	0.21	0.04	0.04	0.20	0.23	0.00	0.26	0.05
		NN1138-2	0.10	0.10	0.00	0.35	0.12	0.04	0.08	0.11
	Glyma.08G110500	N24852	1.48	0.39	0.06	1.05	1.79	0.21	1.49	0.07
		NN1138-2	0.43	0.86	0.32	2.17	0.83	0.68	0.08	1.06
	Glyma.08G110700	N24852	0.70	0.03	0.05	0.32	0.76	0.04	0.68	0.12
		NN1138-2	0.22	0.12	0.06	1.10	0.31	0.30	0.00	0.29
	Glyma.08G110900	N24852	0.07	0.02	0.02	0.12	0.10	0.00	0.02	0.02
		NN1138-2	0.08	0.06	0.06	0.15	0.37	0.13	0.00	0.03
Gm10_LDB_46	Glyma.10G221500	N24852	76.29	26.39	27.96	16.87	27.73	6.97	22.28	143.75
		NN1138-2	19.85	11.59	19.59	11.02	4.60	10.25	16.57	41.63
	Glyma.10g221600	N24852	62.01	90.35	226.97	169.81	181.45	302.06	229.39	94.66
		NN1138-2	146.48	115.01	364.50	284.85	175.95	98.46	356.40	132.33
	Glyma.10g221700	N24852	0.01	0.28	1.47	0.08	0.00	0.01	2.39	0.32
		NN1138-2	0.47	0.97	2.09	1.13	0.01	0.06	2.22	1.10
	Glyma.10g221800	N24852	7.50	6.94	12.51	8.58	5.99	8.60	11.54	15.93
		NN1138-2	6.98	7.15	13.20	11.04	3.23	6.28	9.08	18.26
	Glyma.10g221900	N24852	4.56	7.55	8.59	3.85	2.11	2.66	11.37	7.01
		NN1138-2	8.35	8.73	13.76	8.65	5.75	2.60	11.53	7.97
	Glyma.10g222000	N24852	1.46	0.57	0.25	0.03	0.04	0.07	0.34	0.15

Locus	Gene	Parents	Tissue (FPKM)							
			Leaf	Flower	14seed	21seed	28seed	35seed	7pod	21pod
		NN1138-2	1.03	0.68	0.16	0.14	0.00	0.03	0.67	0.52
	Glyma.10g222200	N24852	2.92	1.14	4.22	3.19	5.30	0.30	3.07	4.56
		NN1138-2	1.59	1.26	3.91	4.82	3.91	4.14	3.46	5.40
	Glyma.10g222300	N24852	10.41	3.42	24.28	7.02	0.73	0.00	27.61	54.47
		NN1138-2	7.30	9.73	48.19	10.98	14.62	3.90	23.50	55.04
	Glyma.10g222400	N24852	1.24	38.37	36.72	85.30	128.53	34.57	210.86	20.40
		NN1138-2	7.99	34.24	2.48	11.54	9.38	16.79	104.31	64.18
	Glyma.10g222500	N24852	1.28	31.04	24.88	1.41	0.06	0.07	140.13	5.76
		NN1138-2	10.01	23.64	334.81	26.65	0.79	0.31	60.73	12.98
	Glyma.10g222600	N24852	3.64	2.10	2.70	0.94	2.30	3.91	3.03	5.55
		NN1138-2	0.63	2.39	2.00	0.76	0.39	0.88	2.08	4.99
	Glyma.10g222800	N24852	4.81	2.85	7.08	5.75	6.13	7.45	10.99	5.75
		NN1138-2	6.45	4.40	17.27	7.05	2.80	5.33	11.07	5.35
	Glyma.10g222900	N24852	0.51	0.58	1.62	1.23	1.82	14.68	2.76	1.41
		NN1138-2	0.47	0.63	3.49	1.74	0.73	0.62	1.87	0.66
	Glyma.10g223100	N24852	0.29	279.04	0.07	0.00	0.06	0.07	0.00	0.00
		NN1138-2	0.07	777.26	0.16	0.00	0.05	0.00	0.13	0.14
	Glyma.10g223200	N24852	0.05	9.15	0.63	0.42	3.23	11.31	0.05	5.10
		NN1138-2	0.00	4.03	0.14	0.22	0.21	0.21	0.11	0.00
	Glyma.10g223300	N24852	5.63	5.92	8.38	4.25	5.19	9.25	5.65	13.21
		NN1138-2	6.60	6.23	7.54	4.82	2.24	2.46	6.45	13.06
	Glyma.10g223400	N24852	32.73	63.53	141.66	96.46	84.66	39.53	188.33	45.16
		NN1138-2	104.09	62.06	216.60	218.55	109.28	68.97	249.42	57.66
	Glyma.10g223500	N24852	4.27	24.42	11.43	0.68	1.17	0.02	8.89	6.46
		NN1138-2	1.53	18.35	2.25	3.30	0.16	0.20	4.09	9.49
	Glyma.10g223600	N24852	0.00	0.00	0.00	0.00	0.00	0.00	0.00	0.00
		NN1138-2	0.00	0.00	0.00	0.37	0.00	0.00	0.00	0.00
	Glyma.10g223700	N24852	0.13	6.14	10.69	11.53	2.08	0.09	29.03	0.03
		NN1138-2	1.64	6.38	20.06	27.23	9.76	6.32	28.53	0.84
	Glyma.10g223800	N24852	2.86	4.94	7.28	1.92	1.30	0.85	9.21	7.07
		NN1138-2	2.05	4.04	9.15	5.55	1.98	1.00	7.81	8.83
	Glyma.10g223900	N24852	0.00	0.22	0.07	0.08	0.00	0.00	0.03	0.06
		NN1138-2	0.03	0.28	0.08	0.24	0.38	0.00	0.00	0.00
	Glyma.10g224000	N24852	2.37	14.65	7.44	3.09	3.02	10.31	9.46	8.45
		NN1138-2	4.05	10.18	17.19	9.17	2.73	2.05	11.30	8.30
	Glyma.10g224100	N24852	1.88	3.16	3.51	1.33	2.24	7.13	2.18	15.53
		NN1138-2	2.80	2.44	5.96	3.08	0.64	2.25	5.08	7.73
	Glyma.10g224200	N24852	7.58	15.20	26.41	15.02	11.05	11.49	34.89	14.18
		NN1138-2	36.21	13.88	42.76	40.28	23.05	11.80	49.12	15.79
	Glyma.10g224300	N24852	3.73	4.09	0.67	0.07	0.05	0.12	0.45	2.85
		NN1138-2	2.98	11.15	1.11	0.85	0.84	0.09	1.83	5.47
	Glyma.10g224400	N24852	0.00	0.04	0.05	0.07	0.10	0.11	0.05	0.05
		NN1138-2	0.39	0.35	0.13	0.00	0.00	0.04	0.22	0.22
	Glyma.10g224500	N24852	7.61	8.23	3.53	1.29	1.83	0.35	7.67	1.75
		NN1138-2	8.06	8.19	6.00	3.53	0.62	1.49	8.54	4.25
	Glyma.10g224600	N24852	0.19	0.43	1.33	0.38	0.50	0.06	1.48	0.58
		NN1138-2	0.57	0.59	1.14	1.96	0.45	0.50	1.60	0.90
	Glyma.10g224700	N24852	0.00	0.15	0.00	0.00	0.00	0.00	0.00	0.00
		NN1138-2	0.00	0.17	0.30	0.00	0.00	0.00	0.00	0.00
	Glyma.10g224900	N24852	10.65	18.88	7.53	3.68	10.60	37.92	11.70	9.42
		NN1138-2	5.93	14.91	10.29	6.09	2.09	2.69	11.87	9.67
	Glyma.10g225000	N24852	0.00	0.00	0.00	0.00	0.00	0.00	0.00	0.00
		NN1138-2	0.00	0.00	0.00	0.00	0.00	0.00	0.00	0.00
	Glyma.10g225100	N24852	5.86	38.56	9.47	1.88	0.74	2.86	5.23	5.32
		NN1138-2	4.54	32.60	17.09	9.91	0.91	0.25	2.91	6.53
Gm12_LDB_16	Glyma.12g073700	N24852	0.12	2.59	5.82	2.12	0.71	0.00	16.62	0.21
		NN1138-2	1.64	1.89	11.86	6.54	0.88	0.46	12.44	0.66
	Glyma.12G073800	N24852	17.42	1.53	3.64	5.37	7.60	1.25	3.94	9.60
		NN1138-2	11.44	3.06	4.17	3.08	2.87	7.10	2.28	6.03
	Glyma.12G073900	N24852	180.77	16.36	23.23	33.09	48.69	19.68	39.38	129.56
		NN1138-2	84.81	28.13	47.24	15.03	33.83	26.62	13.69	29.43
	Glyma.12g074000	N24852	21.62	15.30	10.14	7.20	7.09	5.92	9.92	13.76
		NN1138-2	15.09	18.21	10.90	7.05	3.22	2.99	8.89	8.06
	Glyma.12g074100	N24852	12.17	3.15	7.02	8.10	1.89	0.05	10.66	0.83
		NN1138-2	13.64	7.31	0.12	7.64	5.76	4.23	6.25	3.74
Gm17_LDB_62	Glyma.17G252100	N24852	14.70	17.73	33.15	16.13	16.59	56.94	41.06	17.89
		NN1138-2	18.45	19.20	54.40	42.74	18.40	17.82	47.10	20.72

Locus	Gene	Parents	Tissue (FPKM)							
			Leaf	Flower	14seed	21seed	28seed	35seed	7pod	21pod
	Glyma.17G252400	N24852	2.17	1.53	3.84	0.52	0.17	0.00	18.00	0.18
		NN1138-2	0.91	2.75	2.10	4.85	1.35	0.00	18.88	3.50
	Glyma.17G252600	N24852	29.02	4.65	11.61	3.35	6.79	6.91	17.57	12.74
		NN1138-2	92.22	8.64	7.92	10.99	7.98	5.67	20.21	11.47
	Glyma.17G252700	N24852	2.49	0.39	0.04	0.00	0.00	0.00	0.00	0.00
		NN1138-2	0.00	0.33	0.00	0.00	0.00	0.00	0.04	0.00
	Glyma.17G253000	N24852	5.30	11.79	9.47	4.13	11.31	8.63	5.45	9.51
		NN1138-2	3.90	12.24	9.51	6.68	2.22	4.76	7.70	10.95
	Glyma.17G253100	N24852	11.77	4.98	4.56	4.24	9.14	8.08	4.26	17.28
		NN1138-2	3.96	7.11	2.89	2.17	1.84	3.71	3.80	6.19
	Glyma.17G253200	N24852	0.44	4.11	10.50	4.08	0.39	0.00	16.42	3.19
		NN1138-2	4.77	5.79	15.70	8.17	1.80	0.32	14.40	2.82
	Glyma.17G253300	N24852	15.45	6.06	7.65	2.48	6.24	13.36	6.86	8.93
		NN1138-2	10.87	7.60	7.64	5.79	1.78	2.00	7.71	10.11
	Glyma.17G253400	N24852	1.86	2.17	3.10	3.44	1.24	0.90	3.99	3.69
		NN1138-2	7.43	4.58	4.52	3.77	5.08	1.30	4.18	2.66
	Glyma.17G253500	N24852	3.04	27.93	5.55	0.38	0.00	0.03	2.82	1.29
		NN1138-2	17.91	17.90	0.85	2.15	0.11	0.12	3.44	6.21
	Glyma.17G253600	N24852	3.25	12.93	16.68	11.06	6.73	1.89	14.42	8.20
		NN1138-2	7.64	13.07	18.20	21.88	5.83	7.01	20.27	6.31
	Glyma.17G253700	N24852	1.56	3.79	9.96	3.73	2.38	0.00	13.10	2.65
		NN1138-2	19.21	4.87	13.11	13.04	3.74	3.98	16.70	5.24
	Glyma.17G253900	N24852	54.02	14.42	19.28	6.43	28.13	72.80	12.95	19.42
		NN1138-2	31.73	12.28	8.41	11.39	3.79	6.28	16.40	19.93
Gm04_LDB_41	Glyma.04G165900	N24852	24.91	22.08	13.41	1.88	3.06	1.17	5.59	5.84
		NN1138-2	21.09	11.47	5.91	6.14	1.99	2.55	7.09	12.81
	Glyma.04G166100	N24852	5.53	4.04	13.23	4.86	7.96	6.78	8.97	18.10
		NN1138-2	5.08	5.23	8.86	6.79	1.70	3.08	8.10	16.03
	Glyma.04G166300	N24852	1.86	4.65	8.81	5.47	16.02	32.97	8.81	14.53
		NN1138-2	3.73	4.62	11.84	8.17	4.67	5.47	10.35	10.03
	Glyma.04G166700	N24852	7.57	4.39	9.12	8.62	5.22	2.81	7.20	11.82
		NN1138-2	8.26	7.76	11.74	11.67	13.83	10.53	8.02	13.71
	Glyma.04G166900	N24852	13.14	6.73	5.39	2.94	0.92	0.00	36.01	4.17
		NN1138-2	21.12	12.72	1.80	1.87	1.48	2.21	25.84	11.14
	Glyma.04G167200	N24852	7.53	2.78	5.00	3.86	3.99	0.00	0.26	1.91
		NN1138-2	0.00	2.56	2.85	6.32	5.33	14.77	0.03	0.52
	Glyma.04G167300	N24852	5.44	9.04	4.20	1.69	4.93	9.16	1.11	8.16
		NN1138-2	1.70	8.96	8.16	3.58	1.91	2.87	1.77	9.05
	Glyma.04G167400	N24852	64.92	0.82	3.40	2.87	0.25	0.06	7.41	13.89
		NN1138-2	76.58	4.09	0.45	0.87	0.41	0.12	1.55	2.24
	Glyma.04G167800	N24852	0.54	2.27	2.30	10.30	91.17	1.22	3.11	0.35
		NN1138-2	6.94	6.45	1.45	1.84	5.07	56.21	6.28	1.22
	Glyma.04G167900	N24852	330.30	37.96	67.54	16.98	1.87	0.28	166.90	90.83
		NN1138-2	677.28	68.83	22.22	33.65	19.00	4.47	109.97	118.47
	Glyma.04G168000	N24852	9.92	2.15	1.92	7.54	12.62	7.48	2.26	3.24
		NN1138-2	2.50	1.21	1.05	1.03	0.53	2.11	1.09	0.91
	Glyma.04G168100	N24852	3.75	1.87	1.20	4.32	5.38	3.44	1.21	1.84
		NN1138-2	2.01	2.75	1.69	1.44	1.93	4.30	1.66	0.91
	Glyma.04G168300	N24852	93.06	14.36	8.66	0.74	2.01	0.34	9.12	5.28
		NN1138-2	19.83	17.29	4.72	3.52	0.62	0.85	10.52	16.64
	Glyma.04G168400	N24852	0.00	0.00	0.00	0.00	0.00	0.00	0.00	0.00
		NN1138-2	2.58	1.30	0.00	0.00	0.00	0.00	0.00	0.00
	Glyma.04G168700	N24852	4.21	0.33	0.55	0.20	0.74	1.91	0.80	0.70
		NN1138-2	2.51	0.41	0.36	0.32	0.03	0.30	0.64	1.00
	Glyma.04G169100	N24852	0.90	0.13	1.42	1.50	0.07	0.65	1.33	0.64
		NN1138-2	4.13	0.75	0.72	2.35	1.19	0.78	1.94	1.60
	Glyma.04G169200	N24852	2.34	3.12	4.49	4.18	9.87	18.28	5.95	2.24
		NN1138-2	2.17	2.54	10.77	3.69	4.25	3.17	7.22	2.78
Gm15_LDB_44	Glyma.15G190200	N24852	17.58	17.63	11.82	3.52	5.11	5.53	12.52	17.26
		NN1138-2	8.27	13.49	9.05	9.58	2.00	4.28	14.58	17.66
	Glyma.15G190500	N24852	363.38	247.39	505.60	74.47	119.34	48.99	556.03	665.30
		NN1138-2	455.90	412.12	151.13	97.05	47.04	51.89	579.71	601.10
	Glyma.15G190700	N24852	4.78	5.48	4.62	1.93	5.45	12.38	4.47	6.46
		NN1138-2	2.39	5.28	4.84	4.42	1.87	2.39	4.37	5.49
	Glyma.15G190800	N24852	0.55	1.25	2.15	1.36	2.28	4.21	5.68	0.27
		NN1138-2	2.18	0.65	2.81	1.84	0.23	0.34	2.17	0.57
	Glyma.15G191200	N24852	8.60	21.44	27.52	8.72	8.83	6.72	21.93	28.56

Locus	Gene	Parents	Tissue (FPKM)							
			Leaf	Flower	14seed	21seed	28seed	35seed	7pod	21pod
		NN1138-2	18.91	21.29	20.48	19.00	5.64	7.00	19.29	24.07
	Glyma.15G192100	N24852	0.87	5.95	10.46	4.13	0.73	0.18	30.99	1.04
		NN1138-2	3.31	5.58	28.09	15.35	1.69	1.25	25.08	1.56
	Glyma.15G192300	N24852	6.84	9.05	11.99	3.03	4.85	1.71	11.53	12.75
		NN1138-2	4.90	11.00	8.70	10.81	2.05	4.59	12.02	16.49
	Glyma.15G192700	N24852	1.18	6.17	13.77	3.15	1.57	0.13	40.34	7.53
		NN1138-2	5.03	8.71	26.80	16.50	1.75	1.84	38.05	7.62
	Glyma.15G193000	N24852	3.92	3.17	5.39	2.81	6.42	6.97	9.07	5.48
		NN1138-2	1.66	3.47	11.58	6.72	0.75	2.21	9.93	5.98
	Glyma.15G193100	N24852	6.66	5.90	8.89	4.58	15.62	22.22	8.30	8.27
		NN1138-2	4.97	7.19	9.75	6.81	5.11	6.47	9.89	9.98
	Glyma.15G193300	N24852	2.60	3.37	5.94	3.77	1.53	0.89	9.34	3.42
		NN1138-2	4.45	5.61	11.01	7.04	2.97	1.28	8.23	3.70
	Glyma.15G193400	N24852	2.58	6.54	7.96	2.77	3.27	1.19	8.62	10.17
		NN1138-2	3.37	7.59	10.48	8.60	2.26	3.21	7.91	11.47
	Glyma.15G194000	N24852	11.92	7.49	10.89	4.26	10.37	12.49	10.68	13.73
		NN1138-2	5.01	7.29	11.09	9.54	3.89	5.01	9.38	12.75
	Glyma.15G194300	N24852	182.85	30.13	67.96	18.32	1.17	0.06	101.14	41.72
		NN1138-2	485.22	43.15	15.69	41.48	16.14	6.03	60.96	65.35
	Glyma.15G194600	N24852	19.77	11.89	13.23	9.73	4.47	0.26	7.14	4.67
		NN1138-2	11.24	11.42	2.05	9.52	1.60	3.29	7.33	3.16
	Glyma.15G194900	N24852	2.79	4.47	7.77	4.01	2.05	4.66	10.47	8.31
		NN1138-2	4.93	5.12	20.89	8.86	4.38	2.84	9.09	7.10
	Glyma.15G195000	N24852	2.29	2.18	3.94	1.32	3.05	5.61	5.67	2.42
		NN1138-2	1.26	2.96	7.73	4.69	0.83	1.41	6.34	3.08
	Glyma.15G195100	N24852	1.91	0.72	2.32	1.17	1.49	0.96	1.71	2.88
		NN1138-2	4.05	4.72	10.15	12.18	3.72	6.00	9.51	6.35
	Glyma.15G195200	N24852	0.57	0.73	0.71	1.30	1.02	0.95	2.41	4.77
		NN1138-2	2.44	2.36	4.62	0.65	0.18	0.25	1.28	6.36
	Glyma.15G195500	N24852	29.98	54.29	124.98	87.62	74.02	94.26	141.63	42.09
		NN1138-2	86.43	57.28	201.81	195.10	81.58	62.59	193.22	70.79
	Glyma.15G195600	N24852	0.68	0.98	2.98	0.07	0.00	0.06	0.83	1.38
		NN1138-2	8.01	5.79	1.55	1.51	0.14	0.13	0.79	4.46
	Glyma.15G195700	N24852	28.59	8.53	16.02	16.79	25.25	10.18	16.25	17.66
		NN1138-2	22.88	10.18	11.74	13.24	14.87	16.31	14.10	13.56
	Glyma.15G196400	N24852	6.31	3.44	1.72	0.93	1.30	1.06	2.41	2.04
		NN1138-2	3.63	3.92	2.06	1.58	0.26	0.49	2.56	2.58
	Glyma.15G196800	N24852	23.28	41.21	31.40	11.56	22.59	18.87	29.95	32.96
		NN1138-2	12.26	38.34	43.27	22.25	5.17	8.31	29.66	50.69
	Glyma.15G197100	N24852	0.55	0.14	5.15	0.15	0.00	0.12	2.28	2.10
		NN1138-2	3.15	0.35	1.15	2.13	0.29	0.04	0.90	4.95
	Glyma.15G197200	N24852	6.45	5.00	15.30	6.98	0.98	1.64	17.17	11.28
		NN1138-2	15.23	7.20	4.78	13.20	4.16	2.00	11.59	14.47
	Glyma.15G197800	N24852	1.58	3.07	22.66	9.86	1.88	3.37	14.20	9.77
		NN1138-2	9.87	5.56	10.78	20.02	4.49	2.71	21.30	18.90
	Glyma.15G198100	N24852	13.73	26.90	22.69	22.95	18.34	44.68	21.65	24.73
		NN1138-2	19.12	26.05	38.11	20.86	12.41	12.45	26.81	18.76
	Glyma.15G198300	N24852	8.75	15.39	26.42	13.34	10.09	12.79	20.18	36.36
		NN1138-2	5.52	17.08	31.31	28.23	21.35	14.00	22.16	38.79
	Glyma.15G198600	N24852	8.16	22.29	11.28	3.75	2.81	1.04	14.09	20.78
		NN1138-2	4.86	10.50	14.38	5.02	1.12	1.58	14.01	14.02
	Glyma.15G199700	N24852	4.90	9.00	4.46	0.06	15.32	0.57	9.54	20.16
		NN1138-2	1.82	5.75	0.46	0.55	0.08	0.32	7.59	5.98
	Glyma.15G199800	N24852	17.99	15.00	24.29	15.05	11.05	6.65	13.09	34.75
		NN1138-2	15.52	11.04	20.48	21.16	6.74	7.01	13.51	28.42
	Glyma.15G200800	N24852	29.52	9.79	6.29	1.75	9.80	6.16	5.52	7.09
		NN1138-2	16.94	5.47	4.00	2.81	0.68	1.65	4.93	8.14
	Glyma.15G201100	N24852	6.80	0.95	1.77	2.89	18.84	1.46	2.27	74.08
		NN1138-2	29.63	1.65	6.23	3.30	3.17	10.76	7.49	7.00
	Glyma.15G201200	N24852	9.93	4.51	6.42	3.12	6.92	6.62	7.58	11.26
		NN1138-2	4.22	5.01	9.94	6.58	2.08	3.05	8.12	11.75
	Glyma.15G201300	N24852	13.50	8.85	3.52	1.17	2.15	4.24	4.52	4.66
		NN1138-2	8.09	12.09	1.62	1.65	0.57	0.73	6.31	5.27
	Glyma.15G201400	N24852	2.14	5.05	3.28	1.17	1.32	0.68	3.50	3.80
		NN1138-2	1.71	3.47	1.89	1.68	0.24	0.53	3.05	3.87
	Glyma.15G201500	N24852	4.61	1.64	1.46	0.77	3.90	3.28	1.73	3.82
		NN1138-2	2.34	2.08	1.21	1.08	0.81	0.84	3.49	3.50

Locus	Gene	Parents	Tissue (FPKM)							
			Leaf	Flower	14seed	21seed	28seed	35seed	7pod	21pod
	Glyma.15G202000	N24852	7.00	3.76	12.43	9.63	46.78	36.94	15.20	9.24
		NN1138-2	5.58	6.37	18.31	13.24	3.63	13.48	12.99	5.23
	Glyma.15G202200	N24852	14.02	72.14	13.92	3.52	5.35	20.08	18.74	24.44
		NN1138-2	15.76	46.81	5.64	9.18	1.13	2.43	22.35	15.92
	Glyma.15G202300	N24852	15.49	4.88	3.42	1.86	1.00	1.35	9.79	10.08
		NN1138-2	17.85	5.09	2.05	2.78	0.82	0.87	5.26	4.78
	Glyma.15G202400	N24852	2.26	1.29	1.35	1.31	1.02	1.35	3.72	3.40
		NN1138-2	1.93	1.03	5.03	1.96	0.91	0.83	1.66	2.26
	Glyma.15G202600	N24852	1.52	61.93	1.54	0.29	0.47	1.64	4.06	3.01
		NN1138-2	4.75	121.71	0.64	0.36	0.27	0.24	5.30	2.08
	Glyma.15G202800	N24852	4.53	7.95	19.06	9.68	4.22	1.02	23.73	13.65
		NN1138-2	16.28	9.49	25.86	18.55	8.76	5.69	26.62	13.35
	Glyma.15G203000	N24852	2.47	4.80	17.53	4.47	2.94	0.13	23.78	6.57
		NN1138-2	5.34	4.47	14.19	14.43	1.28	1.16	10.10	3.12
	Glyma.15G203100	N24852	83.73	4.13	15.10	6.16	6.43	0.04	14.95	19.85
		NN1138-2	46.30	6.38	7.04	10.01	4.71	5.72	12.01	14.66
	Glyma.15G203300	N24852	7.21	5.26	12.96	5.76	1.08	0.10	6.51	22.72
		NN1138-2	5.07	6.69	6.50	17.53	3.65	2.95	7.63	6.24
	Glyma.15G203700	N24852	107.52	251.72	75.21	3.21	64.30	346.00	54.07	98.57
		NN1138-2	160.48	327.17	31.02	14.31	4.32	5.15	65.38	224.12
	Glyma.15G203900	N24852	9.07	21.06	27.73	6.94	4.93	0.98	22.93	82.87
		NN1138-2	7.72	28.55	39.39	19.98	8.09	5.22	15.88	64.32
	Glyma.15G204300	N24852	2.82	1.56	4.60	2.64	3.28	2.41	4.97	3.84
		NN1138-2	3.01	2.24	4.92	5.54	1.92	2.53	6.88	2.75
	Glyma.15G205600	N24852	9.01	9.82	11.04	3.86	9.91	18.00	11.48	14.64
		NN1138-2	4.63	9.18	14.55	10.95	3.07	3.87	11.96	13.65
	Glyma.15G205900	N24852	51.58	66.67	48.48	33.69	103.46	291.45	29.39	98.01
		NN1138-2	66.72	58.34	4.86	14.15	14.72	17.76	29.45	81.85
	Glyma.15G206000	N24852	5.12	13.37	18.62	10.17	7.07	7.85	11.11	22.20
		NN1138-2	7.46	10.15	16.55	11.56	8.11	7.33	13.77	19.31
	Glyma.15G206100	N24852	4.98	8.49	7.18	1.31	5.47	8.84	3.29	3.50
		NN1138-2	4.73	5.52	2.57	6.27	0.34	2.57	4.16	13.72
	Glyma.15G206200	N24852	4.44	3.26	3.53	2.59	2.76	5.80	5.34	3.64
		NN1138-2	2.30	3.16	6.74	2.62	0.79	1.00	4.82	2.53
	Glyma.15G207200	N24852	4.76	13.71	20.76	8.74	18.03	32.52	12.64	26.36
		NN1138-2	5.43	17.38	16.22	24.71	3.29	9.99	16.70	19.11
	Glyma.15G207700	N24852	2.62	1.48	2.33	2.32	0.45	0.22	4.42	3.00
		NN1138-2	2.21	2.39	4.49	3.53	0.82	0.93	5.95	7.24
	Glyma.15G208200	N24852	2.38	1.26	3.66	1.14	3.20	2.56	3.64	2.98
		NN1138-2	4.69	2.69	5.60	4.84	1.01	2.28	4.87	3.69
	Glyma.15G208300	N24852	2.62	0.15	1.23	0.00	0.33	0.36	0.31	0.79
		NN1138-2	6.93	0.66	0.87	0.14	1.03	0.00	0.90	0.75
	Glyma.15G209000	N24852	85.71	17.33	39.20	18.66	31.55	36.79	74.69	33.14
		NN1138-2	377.61	27.69	21.47	48.79	11.89	13.23	75.56	47.20
	Glyma.15G209600	N24852	3.49	3.19	6.43	3.51	4.07	2.34	6.59	5.44
		NN1138-2	2.13	4.22	11.60	5.46	2.15	3.04	7.37	6.40
	Glyma.15G209800	N24852	3.48	3.90	5.60	1.74	3.09	1.90	5.12	3.72
		NN1138-2	3.05	4.48	5.92	5.14	0.82	2.03	4.36	4.77
	Glyma.15G209900	N24852	12.55	4.34	6.47	4.21	7.84	15.16	5.78	8.43
		NN1138-2	8.27	5.43	6.63	3.27	2.66	4.00	4.57	6.32
	Glyma.15G210100	N24852	18.58	8.63	2.82	1.10	13.98	1.02	1.50	2.62
		NN1138-2	5.42	6.54	1.49	1.78	1.20	7.08	2.57	4.77
	Glyma.15G210300	N24852	0.97	1.92	5.77	1.63	0.81	0.05	6.64	1.66
		NN1138-2	3.22	3.23	7.00	8.24	0.84	1.67	5.09	2.57
	Glyma.15G211200	N24852	2.34	5.12	2.74	2.44	2.14	2.50	4.67	4.38
		NN1138-2	3.54	4.85	4.61	2.47	0.91	1.74	4.15	3.14
	Glyma.15G211400	N24852	1.83	1.00	1.99	0.42	1.43	1.32	2.59	1.39
		NN1138-2	2.26	0.80	1.76	1.65	0.21	0.44	2.50	1.00
	Glyma.15G211500	N24852	4.88	39.43	0.32	0.23	4.48	9.19	61.50	6.10
		NN1138-2	4.25	12.53	0.27	0.04	0.43	0.07	29.95	2.17
	Glyma.15G211800	N24852	1.64	3.43	1.97	0.97	0.69	0.12	1.64	2.40
		NN1138-2	2.74	3.87	2.39	1.74	0.96	1.34	2.30	2.39
	Glyma.15G211900	N24852	6.63	2.67	4.95	3.53	5.47	11.28	6.14	7.18
		NN1138-2	5.93	4.61	7.79	6.42	1.21	1.94	4.62	4.65
	Glyma.15G212100	N24852	4.53	2.17	3.99	2.81	4.23	8.25	5.73	5.30
		NN1138-2	4.37	3.97	9.55	4.63	1.27	2.04	5.69	4.14
	Glyma.15G212200	N24852	2.36	3.68	6.14	2.39	3.66	5.04	14.16	7.14

Locus	Gene	Parents	Tissue (FPKM)							
			Leaf	Flower	14seed	21seed	28seed	35seed	7pod	21pod
		NN1138-2	1.89	5.06	16.64	7.43	4.39	1.62	13.29	8.29
	Glyma.15G212400	N24852	9.88	13.38	14.87	6.65	12.72	14.89	6.64	15.89
		NN1138-2	5.23	10.25	11.78	9.38	2.93	5.64	7.55	16.02
	Glyma.15G212800	N24852	5.08	4.94	16.72	8.39	7.21	1.35	16.17	9.25
		NN1138-2	3.29	7.61	17.23	20.98	3.57	7.40	16.65	11.80
	Glyma.15G213100	N24852	15.70	9.10	11.46	4.72	2.91	2.54	14.48	11.46
		NN1138-2	18.75	8.34	9.24	11.89	2.22	2.32	12.95	7.48
	Glyma.15G213200	N24852	6.16	6.54	12.55	4.39	8.06	8.39	14.10	10.94
		NN1138-2	6.53	7.93	13.85	12.88	3.49	5.41	15.58	11.88
	Glyma.15G213400	N24852	5.19	4.97	7.18	3.15	9.73	14.27	4.24	11.82
		NN1138-2	3.05	4.66	6.12	5.75	1.91	5.61	5.09	7.40
	Glyma.15G213600	N24852	0.21	0.05	0.09	0.00	0.00	0.00	0.05	0.34
		NN1138-2	17.39	0.08	0.00	0.00	0.00	0.00	0.00	0.09
	Glyma.15G213700	N24852	22.93	2.82	5.50	0.07	0.07	0.02	9.43	16.97
		NN1138-2	13.86	4.56	0.73	0.56	0.23	0.83	6.53	14.18
	Glyma.15G214500	N24852	2.79	3.84	2.97	1.28	2.12	3.34	3.14	4.59
		NN1138-2	1.34	4.09	4.85	3.26	0.65	1.21	3.04	4.11
	Glyma.15G215000	N24852	10.43	11.08	1.99	0.32	1.96	2.38	0.81	4.94
		NN1138-2	4.98	15.55	0.73	1.34	1.00	0.96	1.81	7.29
	Glyma.15G215100	N24852	16.96	26.77	4.39	5.22	4.69	3.06	4.71	10.49
		NN1138-2	12.09	22.01	9.95	4.44	1.91	3.45	3.42	4.23
	Glyma.15G215600	N24852	0.92	3.36	28.41	22.65	6.05	0.03	26.86	0.07
		NN1138-2	12.22	5.15	26.77	40.15	7.46	8.33	27.78	8.11
	Glyma.15G215900	N24852	18.74	4.89	4.51	4.75	2.25	0.60	5.17	6.33
		NN1138-2	18.34	4.30	3.97	3.57	1.51	4.35	3.39	2.33
	Glyma.15G216000	N24852	2.03	1.19	3.48	2.38	3.63	4.97	3.96	4.60
		NN1138-2	3.17	2.10	6.22	5.56	1.46	2.09	5.31	7.98
	Glyma.15G216100	N24852	3.44	1.13	2.98	1.41	2.94	1.69	5.51	4.63
		NN1138-2	2.27	2.57	4.73	3.95	0.96	0.94	3.62	5.59
	Glyma.15G216400	N24852	7.51	3.61	7.97	4.95	8.63	21.70	5.85	9.29
		NN1138-2	6.97	5.05	9.30	8.50	3.27	4.01	6.69	7.64
	Glyma.15G216700	N24852	0.45	0.79	2.86	0.10	0.03	0.00	0.57	1.62
		NN1138-2	2.82	3.95	0.20	1.26	0.00	0.00	0.44	4.82
	Glyma.15G217100	N24852	0.26	102.85	194.65	0.15	0.33	0.12	109.15	264.27
		NN1138-2	3.98	87.53	5.14	6.69	0.73	0.84	128.67	476.80
	Glyma.15G217300	N24852	0.32	2.86	2.42	0.46	0.10	0.05	10.02	0.11
		NN1138-2	3.99	3.00	1.98	1.27	0.06	0.04	3.46	0.82
	Glyma.15G217400	N24852	4.65	3.12	12.40	2.84	5.10	8.15	10.04	8.01
		NN1138-2	3.50	4.14	13.33	10.10	2.66	4.54	10.48	12.50
	Glyma.15G217500	N24852	1.13	3.06	37.68	73.06	17.00	2.79	6.33	2.26
		NN1138-2	1.21	2.65	11.88	86.42	65.95	25.77	7.45	1.29
	Glyma.15G217700	N24852	12.36	6.77	15.64	18.35	26.88	56.53	11.45	35.76
		NN1138-2	8.22	12.99	11.81	22.35	188.65	44.94	9.30	48.07
	Glyma.15G218000	N24852	6.55	7.00	10.53	6.09	5.62	1.80	11.46	8.07
		NN1138-2	12.83	10.07	14.19	10.66	4.83	5.49	10.97	7.50
	Glyma.15G218200	N24852	2.63	13.21	8.81	1.97	2.17	0.70	6.16	8.77
		NN1138-2	4.35	15.24	4.65	5.24	1.29	1.84	4.77	6.84
	Glyma.15G218400	N24852	1.68	53.34	16.81	3.59	1.37	0.88	7.90	7.44
		NN1138-2	8.22	47.75	26.11	14.52	2.91	2.17	8.63	7.81
	Glyma.15G219100	N24852	7.25	9.26	24.44	5.53	1.30	0.05	22.27	28.66
		NN1138-2	11.98	14.37	13.26	17.98	3.13	3.92	22.35	36.07
	Glyma.15G219200	N24852	7.22	7.58	9.59	2.99	7.43	10.74	9.30	8.84
		NN1138-2	4.70	7.35	13.28	9.99	1.31	4.83	10.17	10.39
	Glyma.15G219300	N24852	4.53	4.65	9.81	1.42	3.64	1.40	8.78	23.35
		NN1138-2	2.85	7.19	9.33	7.68	1.11	1.06	7.39	14.15
	Glyma.15G219400	N24852	1.52	3.31	2.68	1.53	2.54	0.05	0.23	2.88
		NN1138-2	9.00	1.76	0.95	2.98	0.90	2.32	0.23	1.77
	Glyma.15G220600	N24852	0.82	0.81	0.44	0.22	0.22	0.23	0.95	0.45
		NN1138-2	2.99	2.23	6.30	2.47	1.11	1.35	3.89	2.42
	Glyma.15G220800	N24852	0.52	1.32	3.45	0.20	0.70	0.24	0.97	1.44
		NN1138-2	10.01	6.46	1.46	1.89	0.39	0.96	1.97	10.49
	Glyma.15G221300	N24852	21.13	148.53	40.66	9.88	20.63	0.13	165.97	6.03
		NN1138-2	133.21	121.38	15.14	28.25	9.58	7.87	160.57	33.45
Gm16_LDB_1	Glyma.16G000200	N24852	3.67	8.82	21.48	4.19	6.57	0.30	19.76	18.41
		NN1138-2	9.62	13.81	13.67	14.46	1.27	2.25	23.52	17.65
	Glyma.16G000300	N24852	11.62	8.82	15.56	10.68	25.47	50.24	16.50	16.93
		NN1138-2	9.56	11.60	18.94	15.14	5.85	10.10	14.94	15.02

Locus	Gene	Parents	Tissue (FPKM)							
			Leaf	Flower	14seed	21seed	28seed	35seed	7pod	21pod
	Glyma.16G000500	N24852	10.55	9.05	11.62	3.74	8.12	8.40	11.33	11.94
		NN1138-2	8.81	10.58	16.53	12.63	4.05	4.96	13.56	13.54
	Glyma.16G000700	N24852	5.82	2.54	2.19	2.32	0.92	1.65	5.49	7.32
		NN1138-2	1.32	2.07	3.23	1.23	0.20	0.22	1.31	2.10
	Glyma.16G000800	N24852	2.16	0.80	0.67	0.53	0.56	0.44	1.80	2.67
		NN1138-2	0.64	0.80	1.48	0.31	0.62	0.05	0.50	0.64
	Glyma.16G001000	N24852	4.24	3.42	5.72	2.09	6.96	6.83	7.55	8.07
		NN1138-2	1.11	2.84	10.37	6.82	0.53	1.87	8.68	7.47
	Glyma.16G001300	N24852	3.30	10.64	8.41	5.90	6.03	2.75	6.50	5.66
		NN1138-2	2.88	7.20	8.31	8.72	3.43	4.76	4.51	8.52
	Glyma.16G001600	N24852	0.97	3.78	12.45	0.33	0.12	0.74	2.65	14.92
		NN1138-2	2.26	0.74	2.04	1.15	0.33	0.40	1.27	2.32
	Glyma.16G001700	N24852	7.72	7.56	17.86	11.71	23.94	21.76	15.56	23.62
		NN1138-2	9.16	8.12	17.35	13.37	6.52	14.99	14.48	16.54
	Glyma.16G001800	N24852	7.98	14.55	30.58	9.23	8.58	1.92	19.37	17.13
		NN1138-2	8.18	14.04	21.08	33.11	10.80	14.74	19.31	29.09
	Glyma.16G001900	N24852	48.30	52.27	123.72	90.78	64.96	42.32	151.72	70.09
		NN1138-2	73.10	51.17	161.38	162.59	91.81	60.92	153.73	63.91
	Glyma.16G002300	N24852	16.02	17.23	16.81	7.29	12.50	15.57	15.21	16.36
		NN1138-2	13.11	15.30	15.81	12.96	3.79	8.24	14.84	15.38
	Glyma.16G002400	N24852	12.67	18.87	24.50	19.37	11.22	2.47	21.38	28.82
		NN1138-2	11.39	20.80	22.82	17.17	6.24	13.03	23.62	22.39
	Glyma.16G002500	N24852	336.60	113.80	53.03	41.00	71.18	150.32	135.43	144.18
		NN1138-2	217.99	137.43	136.33	48.83	32.21	19.55	118.56	41.83
	Glyma.16G002700	N24852	2.04	2.36	6.58	1.50	1.12	1.17	2.39	11.45
		NN1138-2	0.58	1.48	0.67	1.13	0.41	0.99	0.66	3.73
	Glyma.16G003000	N24852	12.00	14.28	19.73	8.22	21.19	28.94	16.82	22.67
		NN1138-2	7.55	12.80	17.79	17.18	5.43	9.59	21.19	24.52
	Glyma.16G003100	N24852	1.34	2.88	5.13	3.10	3.02	1.34	7.95	4.60
		NN1138-2	3.93	2.98	9.38	6.52	2.41	2.71	7.23	3.25
	Glyma.16G003200	N24852	4.57	4.44	7.04	2.02	3.00	2.22	9.21	9.14
		NN1138-2	3.23	7.23	9.10	5.37	1.10	1.70	10.43	8.68
	Glyma.16G003300	N24852	3.88	1.85	5.57	1.55	2.36	0.83	8.88	5.68
		NN1138-2	3.39	3.21	6.77	5.82	0.92	1.51	9.38	7.89
	Glyma.16G003600	N24852	7.02	14.52	16.42	6.47	7.07	4.26	17.78	15.67
		NN1138-2	5.13	16.58	23.00	23.49	7.65	7.33	19.14	14.85
	Glyma.16G003700	N24852	9.36	7.18	12.75	5.59	13.72	12.72	18.43	18.89
		NN1138-2	7.47	11.70	22.83	14.58	3.54	7.24	23.36	18.86
	Glyma.16G003900	N24852	8.97	9.70	11.42	2.38	5.30	8.13	12.05	9.55
		NN1138-2	7.38	8.33	8.15	9.58	1.21	3.62	14.22	13.40
	Glyma.16G004000	N24852	6.73	8.44	8.36	1.71	3.30	6.73	8.82	7.80
		NN1138-2	5.02	7.86	5.66	8.48	0.46	2.98	10.10	9.81
	Glyma.16G004200	N24852	6.37	11.78	9.48	4.01	4.70	3.42	7.10	9.77
		NN1138-2	5.00	10.25	7.30	7.64	1.88	5.65	7.02	8.97
	Glyma.16G004300	N24852	6.75	1.31	6.18	0.56	1.17	0.26	12.30	4.82
		NN1138-2	2.25	1.91	2.95	3.25	0.86	1.80	5.99	14.02
	Glyma.16G004400	N24852	16.38	39.07	36.95	12.08	12.89	3.79	33.75	28.29
		NN1138-2	20.52	36.46	32.39	45.91	10.45	14.30	33.84	28.95
	Glyma.16G004800	N24852	4.95	1.60	5.21	1.66	9.68	12.93	5.61	6.57
		NN1138-2	0.77	1.96	5.96	4.13	0.58	1.76	8.83	8.13
	Glyma.16G005100	N24852	10.93	1.12	1.27	0.00	0.00	0.03	2.17	0.49
		NN1138-2	0.59	0.07	0.02	0.01	0.00	0.00	0.05	0.01
	Glyma.16G005200	N24852	3.54	6.60	16.90	11.27	8.53	2.94	13.47	9.18
		NN1138-2	4.44	12.20	12.38	13.47	3.91	3.96	20.40	14.05
	Glyma.16G005300	N24852	9.69	0.32	1.25	0.13	0.05	0.05	1.67	1.81
		NN1138-2	15.96	0.34	0.38	0.16	0.06	0.02	1.51	0.92
	Glyma.16G005400	N24852	6.73	7.58	11.27	9.59	16.46	16.90	14.14	5.86
		NN1138-2	7.04	9.56	22.27	13.79	8.77	7.16	15.66	4.58
	Glyma.16G005600	N24852	5.53	4.34	4.37	1.82	5.02	5.06	5.04	6.21
		NN1138-2	3.30	3.93	5.77	4.11	0.53	1.88	5.49	4.67
	Glyma.16G005700	N24852	6.88	9.85	11.18	4.45	6.56	6.77	10.81	12.64
		NN1138-2	4.36	9.13	10.50	8.63	0.50	4.72	9.88	10.80
	Glyma.16G005800	N24852	9.33	18.44	5.72	0.31	0.34	1.13	17.18	9.76
		NN1138-2	15.97	17.60	3.16	1.72	0.22	0.14	14.12	11.58
	Glyma.16G006000	N24852	6.42	5.32	8.60	4.48	10.00	15.22	12.43	10.19
		NN1138-2	3.18	5.66	18.29	8.59	1.98	3.61	11.91	7.72
	Glyma.16G006100	N24852	4.98	2.37	9.45	1.27	1.27	0.33	5.85	6.37

Locus	Gene	Parents	Tissue (FPKM)							
			Leaf	Flower	14seed	21seed	28seed	35seed	7pod	21pod
		NN1138-2	0.60	3.85	6.47	4.25	0.64	0.74	5.71	16.14

FPKM, Fragments Per Kilobase Million. In Tissue column, Leaf, collected before flowering; Flower, collected at flowering;14seed, 21seed, 28seed and 35seed, the seeds at14, 21, 28, 35 days after flowering, respectively; 7pod and 21pod, the pods at 7 and 21 days after flowering, respectively.

The shaded rows indicate the SCC and DTF candidate genes identified in the present study.

Table S7 The allelic variations of candidate genes between *N24852* and *NN1138-2*

Gene	Description	Variant site	Allele variant		
			1. W	2. C	3. Variant
Glyma.01G194500	ADP-ribosylation factor-like A1C	-			
Glyma.01G194600	Homeodomain-like DNA-binding family	-			
Glyma.01G194700	N-acetyl-l-glutamate synthase 1	-			
Glyma.01G194800	Uncharacterized conserved protein	52896720	A	C	Missense variant
		52896721	T	A	Missense variant
Glyma.01G194900	heptahelical protein 4	-			
Glyma.01G195000	Galactosyl transferase GMA12 family protein	-			
Glyma.01G195100	-	52926362	C	A	Missense variant
		52926987	A	G	Stop_gain
Glyma.01G195200	ARM repeat superfamily protein	52942422	G	C	Missense variant
Glyma.01G195400	BSD domain-containing protein	-			
Glyma.01G195500	ACT domain repeat 1	-			
Glyma.01G195600	Carbohydrate-binding protein	52968397	G	A	Missense variant
Glyma.01G195700	phytosulfokine 4 precursor	-			
Glyma.01G195900	Integrase-type DNA-binding superfamily	-			
Glyma.01G196000	receptor like protein 44	-			
Glyma.01G196300	branched-chain aminotransferase 3	53064207	G	C	Missense variant
Glyma.01G196600	Homeobox-leucine zipper protein family	-			
Glyma.01G196800	xyloglucan endotransglucosylase/hydrolase 6	53108732	C	G	Missense variant
Glyma.01G197100	nitrogen fixation S (NIFS)-like 1	-			
Glyma.01G197200	-	-			
Glyma.01G197300	-	-			
Glyma.01G197500	tubulin alpha-2 chain	-			
Glyma.01G197600	HAESA-like 2	-			
Glyma.01G197700	peroxisomal NAD-malate dehydrogenase 1	-			
Glyma.01G197800	Leucine-rich receptor-like protein kinase	-			
Glyma.01G198100	bHLH DNA-binding superfamily protein	-			
Glyma.01G198200	-	-			
Glyma.01G198400	-	53219247	C	A	Missense variant
		53219296	G	A	Missense variant
		53219304	A	C	Missense variant
Glyma.01G198500	-	53229579	G	A	Stop_gain
Glyma.01G198600	Peptidase M20/M25/M40 family protein	-			
Glyma.01G198900	AT hook motif DNA-binding family protein	-			
Glyma.01G199000	Clathrin adaptor complexes medium subunit	53297341	T	C	Stop_gain
Glyma.01G199100	-	-			
Glyma.01G199200	lsd one like 1	53308610	G	T	Missense variant
Glyma.01G199300	-	53311555	G	C	Missense variant
Glyma.01G199400	aluminum-activated, malate transporter 12	53322135	A	G	Missense variant
Glyma.01G199600	poltergeist like 1	53333405	T	A	Stop_gain
		53334468	T	C	Missense variant
		53334898	G	A	Missense variant
		53335306	C	G	Missense variant
		53335309	T	C	Missense variant
Glyma.01G199700	glycine decarboxylase complex H	53348097	G	A	Missense variant
Glyma.01G199900	-	-			
Glyma.01G200000	-	-			
Glyma.01G200100	nitrate transporter 1.5	-			
Glyma.01G200200	plastidial pyruvate kinase 3	53396771	C	A	Missense variant
		53396860	T	C	Missense variant
		53397586	G	T	Missense variant
Glyma.01G200500	myo-inositol polyphosphate 5-phosphatase 2	-			
Glyma.01G200600	ovate family protein 13	53423278	G	T	Missense variant
Glyma.01G200700	tobamovirus multiplication 2A	-			
Glyma.01G200800	CheY-like two-component responsive	53440513	C	A	Missense variant
		53440728	C	T	Missense variant
Glyma.01G200900	-	-			
Glyma.01G201000	plastid ribosomal protein l11	-			

Gene	Description	Variant site	Allele variant		
			1. W	2. C	3. Variant
Glyma.01G201100	Vacuolar protein sorting 55 (VPS55) family	-			
Glyma.01G201200	-	-			
Glyma.01G201300	phosphoglucosamine mutase-related	-			
Glyma.01G201400	phosphoglucosamine mutase-related	-			
Glyma.01G202000	HAD superfamily protein	-			
Glyma.08G109200	Chalcone and stilbene synthase family protein	-			
Glyma.08G109300	Chalcone and stilbene synthase family protein	-			
Glyma.08G109400	Chalcone and stilbene synthase family protein	8392292	T	C	5_prime_UTR_varia
		8392915	G	A	Missense variant
Glyma.08G109500	Chalcone and stilbene synthase family protein	-			
Glyma.08G109600	Chalcone and stilbene synthase family protein	-			
Glyma.08G110300	Chalcone and stilbene synthase family protein	-			
Glyma.08G110400	Chalcone and stilbene synthase family protein	-			
Glyma.08G110500	Chalcone and stilbene synthase family protein	-			
Glyma.08G110700	Chalcone and stilbene synthase family protein	-			
Glyma.08G110900	Chalcone and stilbene synthase family protein	-			
Glyma.10G221500	Gigantea protein (GI)	45310798	A	T	Stop_gain
Glyma.10G221600	Ribosomal protein S13/S18 family	-			
Glyma.10G221700	Nucleotide-sugar transporter family protein	-			
Glyma.10G221800	Uncharacterised conserved protein	45329450	G	A	Missense variant
Glyma.10G221900	-	-			
Glyma.10G222000	-	45348384	C	G	Missense variant
Glyma.10G222200	Protein kinase superfamily protein	-			
Glyma.10G222300	Protein of unknown function (DUF3511)	-			
Glyma.10G222400	Peroxidase superfamily protein	45366573	A	T	Missense variant
Glyma.10G222500	Peroxidase superfamily protein	45374068	C	A	Missense variant
Glyma.10G222600	Protein kinase superfamily protein	45379502	C	G	Missense variant
Glyma.10G222800	SET domain group 26	-			
Glyma.10G222900	DHHC-type zinc finger family protein	-			
Glyma.10G223000	Plant invertase inhibitor superfamily protein	-			
Glyma.10G223100	-	-			
Glyma.10G223200	Integrase-type DNA-binding superfamily	-			
Glyma.10G223300	polypyrimidine tract-binding protein 3	-			
Glyma.10G223400	ribosomal protein 1	-			
Glyma.10G223500	cellulose synthase 6	-			
Glyma.10G223600	Protein of unknown function (DUF1278)	-			
Glyma.10G223700	Cupredoxin superfamily protein	-			
Glyma.10G223800	bZIP transcription factor family protein	-			
Glyma.10G223900	Protein of unknown function (DUF677)	-			
Glyma.10G224100	thioredoxin Y1	45494542	C	T	Missense variant
Glyma.10G224200	Ribosomal protein S13/S18 family	45499621	C	T	Missense variant
		45499683	C	G	Missense variant
		45499740	A	G	Missense variant
		45499753	T	A	Missense variant
		45499763	A	C	Missense variant
		45499769	A	C	Missense variant
Glyma.10G224300	-	45503767	C	G	Missense variant
		45504151	T	A	Missense variant
		45504168	C	G	Missense variant
Glyma.10G224400	HSP20-like chaperones superfamily protein	45509578	G	C	Missense variant
		45510378	T	A	Missense variant
Glyma.10G224500	Protein of unknown function (DUF793)	45515095	A	C	Missense variant
Glyma.10G224600	-	-			
Glyma.10G224700	-	-			
Glyma.10G224900	tubby like protein 10	-			
Glyma.10G225000	-	-			
Glyma.10G225100	Duplicated homeodomain-like superfamily	45563143	T	C	Missense variant
Glyma.12g073700	Protein kinase superfamily protein	-			
Glyma.12G073800	-	-			

Gene	Description	Variant site	Allele variant		
			1. W	2. C	3. Variant
Glyma.12G073900	Gseudo-response regulator 3	5519728	C	A	Missense variant
		5520945	C	T	Stop_gain
Glyma.12g074000	Protein kinase superfamily protein	-			
Glyma.12g074100	phototropin 1	5545908	C	T	Missense variant
		5557212	C	T	Missense variant
Glyma.17G252100	Proteasome alpha subunit F1	40649315	C	G	Missense variant
Glyma.17G252600	Regulatory particle triple-A ATPase 4A	-			
Glyma.17G253000	Sec14p-like phosphatidylinositol transfer	40712203	T	G	Start_codon_gain_va
Glyma.17G253100	Bifunctional nuclease in basal defense	40716316	C	T	Missense variant
Glyma.17G253200	Basic leucine-zipper 44	-			
Glyma.17G253300	RING/U-box superfamily protein	-			
Glyma.17G253400	NAD(P) superfamily protein	-			
Glyma.17G253500	TLC Lipid-sensing domain containing protein	40754616	T	A	5_prime_UTR_varia
		40755078	G	A	Missense variant
		40755383	A	G	Missense variant
Glyma.17G253600	Regulatory particle triple-A ATPase 4A	-			
Glyma.17G253700	UDP-Glycosyltransferase superfamily protein	40762395	C	T	Missense variant
		40766099	A	T	Missense variant
Glyma.17G253900	GTP-binding protein, HflX	-			
Glyma.04G165900	zinc fingerfamily protein	-			
Glyma.04G166100	ATPase E1-E2 type family protein	-			
Glyma.04G166300	CCT motif-containing response regulator	-			
Glyma.04G166700	FKBP-like peptidyl-prolyl cis-trans isomerase	-			
Glyma.04G166900	myb domain protein 16	-			
Glyma.04G167200	NAC domain containing protein 100	-			
Glyma.04G167400	NDH dependent flow 6	42009788	T	G	Missense variant
Glyma.04G167800	expansin A15	-			
Glyma.04G167900	light-harvesting chlorophyll-protein complex I				
Glyma.04G168000	-	42173844	A	C	Missense variant
		42173926	G	A	Missense variant
		42174010	A	T	Missense variant
Glyma.04G168100	nicotinate phosphoribosyltransferase 2	-			
Glyma.04G168300	cycling DOF factor 3	-			
Glyma.04G168400	-	-			
Glyma.04G168700	Small GTP-binding protein	-			
Glyma.04G169100	-	-			
Glyma.04G169200	Protein of unknown function (DUF3223)	42358749	C	G	Missense variant
Glyma.15G190200	squamosa promoter-binding protein-like 12	20247886	A	G	Missense variant
		20250747	A	C	Missense variant
		20250795	A	G	Missense variant
		20250871	G	A	Missense variant
		20250957	G	A	Missense variant
		20256490	G	T	Missense variant
		20257718	T	G	Missense variant
Glyma.15G190500	S-adenosylmethionine synthetase family	-			
Glyma.15G190700	ferric reductase transmembrane protein family	-			
Glyma.15G190800	Subtilase family protein	20462031	T	G	Missense-variant
Glyma.15G191200	gamma-soluble NSF attachment protein	-			
Glyma.15G192100	SNF7 family protein	-			
Glyma.15G192300	VPS54	20930636	A	C	Missense-variant
Glyma.15G192700	Outer arm dynein light chain 1 protein	-			
Glyma.15G193000	periodic tryptophan protein 2	21116051	C	G	Missense-variant
Glyma.15G193100	RNA-binding family protein	21175332	A	G	Missense-variant
		21178059	T	G	Missense-variant
Glyma.15G193300	Phototropic-responsive NPH3 family protein	-			
Glyma.15G193400	cation-chloride co-transporter 1	21274732	T	C	Missense-variant
Glyma.15G194000	binding;RNA binding	21478526	A	C	Missense-variant
		21481576	G	C	Missense-variant
Glyma.15G194300	photosystem I subunit K	-			

Gene	Description	Variant site	Allele variant		
			1. W	2. C	3. Variant
Glyma.15G194600	inositol transporter 2	-			
Glyma.15G194900	endoplasmic reticulum oxidoreductins 2	-			
Glyma.15G195000	Transducin/WD40 repeat-like superfamily	-			
Glyma.15G195100	-	-			
Glyma.15G195200	fatty acid desaturase 2	-			
Glyma.15G195500	Ribosomal protein L32e	-			
Glyma.15G195600	AWPM-19-like family protein	-			
Glyma.15G195700	-	-			
Glyma.15G196400	SCP1-like small phosphatase 4	22619508	T	C	Missense-variant
Glyma.15G196800	MEI2-like protein 5	-			
Glyma.15G197100	Chaperone DnaJ-domain superfamily protein	22898554	G	A	Missense-variant
Glyma.15G197200	Mitochondrial transcription termination factor	-			
Glyma.15G197800	glucuronidase 3	23128512	C	A	Missense-variant
		23130392	T	C	Missense-variant
		23130427	T	A	Missense-variant
Glyma.15G198100	-	-			
Glyma.15G198300	UBA domain-containing protein	-			
Glyma.15G198600	Major facilitator superfamily protein	23414486	G	A	Stop_lost
		23414674	T	C	Missense-variant
		23414917	G	A	Missense-variant
		23416130	G	C	Missense-variant
Glyma.15G199700	-	23940382	C	G	Missense-variant
		23940805	A	C	Missense-variant
Glyma.15G199800	-	-			
Glyma.15G200800	S-adenosyl-L-methionine-dependent	24530327	G	T	Missense-variant
Glyma.15G201100	flavin-dependent monooxygenase 1	-			
Glyma.15G201200	Dihydroxyacetone kinase	24692047	G	A	Missense-variant
		24692065	G	T	Missense-variant
		24695739	G	C	Missense-variant
		24697636	G	A	Missense-variant
Glyma.15G201300	DNA-binding protein phosphatase 1	24737802	A	T	Missense-variant
Glyma.15G201400	Major facilitator superfamily protein	-			
Glyma.15G201500	-	-			
Glyma.15G202000	Phox/Bem1p domain	24919863	A	T	Missense-variant
		24921654	C	A	Missense-variant
Glyma.15G202200	KNOTTED1-like homeobox gene 3	25222812	T	A	Missense-variant
Glyma.15G202300	Cyclophilin-like peptidyl-prolyl cis-trans	-			
Glyma.15G202400	-	-			
Glyma.15G202600	-	-			
Glyma.15G202800	Ribosomal protein L39 family protein	-			
Glyma.15G203000	Endomembrane protein 70 protein family	25783552	T	G	Missense-variant
		25784877	C	A	Missense-variant
Glyma.15G203100	adenylate kinase family protein	-			
Glyma.15G203300	sugar transporter protein 7	25841624	A	G	Missense-variant
Glyma.15G203700	SOS3-interacting protein 3	-			
Glyma.15G203900	LJRHL1-like 1	26407880	C	A	Missense-variant
Glyma.15G204300	Surfeit locus protein 5 subunit 22 of Mediator	26956594	G	C	Missense-variant
Glyma.15G205600	homolog of yeast FIP1 [V]	27805583	A	G	Missense-variant
Glyma.15G205900	temperature-induced lipocalin	-			
Glyma.15G206000	Protein of unknown function (DUF1068)	-			
Glyma.15G206100	Calmodulin-binding protein	-			
Glyma.15G206200	response regulator 12	28960244	T	C	Missense-variant
Glyma.15G207200	acyl-activating enzyme 17	-			
Glyma.15G207700	Protein of unknown function (DUF1637)	29728740	A	G	Missense-variant
Glyma.15G208200	FAD-dependent oxidoreductase family protein	29881792	T	C	Missense-variant
Glyma.15G208300	photosystem II reaction center protein M	-			
Glyma.15G209000	Ribosomal protein L13 family protein	-			
Glyma.15G209600	RING/U-box superfamily protein	-			
Glyma.15G209800	BED zinc finger ;hAT family dimerisation	-			

Gene	Description	Variant site	Allele variant		
			1. W	2. C	3. Variant
Glyma.15G209900	NADH:ubiquinone oxidoreductase	-			
Glyma.15G210100	trehalose-6-phosphate synthase	32196229	A	C	Start_codon_gain_va
Glyma.15G210300	rhamnose biosynthesis 1	-			
Glyma.15G211200	Protein kinase superfamily protein	-			
Glyma.15G211400	recA DNA recombination family protein	32648953	T	A	Missense-variant
Glyma.15G211500	protease inhibitor protein	-			
Glyma.15G211800	Nodulin MtN3 family protein	-			
Glyma.15G211900	SH2 domain protein A	32886879	G	C	Missense-variant
Glyma.15G212100	SH2 domain protein B	-			
Glyma.15G212200	-	-			
Glyma.15G212400	LEM3 family protein	-			
Glyma.15G212800	GTPase binding;GTP binding	33450509	T	C	Missense variant&
Glyma.15G213100	short-chain dehydrogenase-reductase B	-			
Glyma.15G213200	cyclophilin 59	-			
Glyma.15G213400	Phox/Bem1p domain	-			
Glyma.15G213600	S-locus lectin protein kinase family protein	34071178	G	A	Missense variant&
Glyma.15G213700	S-locus lectin protein kinase family protein	-			
Glyma.15G214500	-	34522282	G	T	Missense-variant
Glyma.15G215000	Homeodomain-like superfamily protein	34983211	C	G	Missense-variant
Glyma.15G215100	bHLH superfamily protein	35032013	T	C	Missense-variant
Glyma.15G215600	Leucine-rich repeat protein kinase family	-			
Glyma.15G215900	NYC1-like	-			
Glyma.15G216000	DEA(D/H)-box RNA helicase family protein	35292443	A	G	Missense-variant
		35292617	T	C	Missense-variant
		35292647	A	G	Missense-variant
Glyma.15G216100	DEA(D/H)-box RNA helicase family protein	35392977	T	C	Missense-variant
Glyma.15G216400	ribonuclease Ps	35591225	C	T	Missense-variant
		35592964	A	T	Missense-variant
		35594412	A	C	Missense-variant
Glyma.15G216700	HSP20-like chaperones superfamily protein	-			
Glyma.15G217100	MLP-like protein 43	-			
Glyma.15G217300	DNA glycosylase superfamily protein	-			
Glyma.15G217400	floral homeotic protein (HUA1)	-			
Glyma.15G217700	Calcium-binding EF-hand family protein	-			
Glyma.15G218000	Protein of unknown function (DUF1138)	-			
Glyma.15G218200	Protein kinase superfamily protein	-			
Glyma.15G218400	HAD superfamily	-			
Glyma.15G219100	metacaspase 4	37775669	A	T	Missense-variant
		37776033	C	T	Missense-variant
		37778251	C	T	Missense-variant
Glyma.15G219200	RING/FYVE/PHD zinc finger superfamily	-			
Glyma.15G219300	Ca(2)-dependent phospholipid-binding protein	38021598	T	C	Missense-variant
		38032903	A	C	Missense-variant
Glyma.15G219400	Ca(2)-dependent phospholipid-binding protein	38166920	A	G	Missense-variant
Glyma.15G220600	F-box and associated interaction domains-	39770397	A	G	Missense-variant
Glyma.15G220800	NADH:cytochrome B5 reductase 1	-			
Glyma.15G221300	UDP-glucosyl transferase 73B3	39953287	T	G	Missense-variant
Glyma.16G000200	methionine S-methyltransferase	21145	A	G	Missense variant
		21270	T	G	Missense variant
Glyma.16G000300	auxin response factor 1	27033	C	G	Missense variant
Glyma.16G000500	EMBRYO DEFECTIVE 140	44218	T	C	Missense variant
		55643	A	G	Missense variant
Glyma.16G000700	Core-2/I-branching beta-1,6-N-	-			
Glyma.16G000800	Protein of unknown function (DUF707)				
Glyma.16G001000	glutamine-rich protein 23	68705	A	G	Missense variant
		69170	C	A	Missense variant
Glyma.16G001300	-	-			
Glyma.16G001600	Protein of unknown function (DUF579)	95565	C	T	Missense variant
Glyma.16G001700	Ras-related small GTP-binding family protein	-			

Gene	Description	Variant site	Allele variant		
			1. W	2. C	3. Variant
Glyma.16G001800	RAB GTPase 11C	-			
Glyma.16G001900	Translation elongation factor EF1B, gamma	-			
Glyma.16G002300	Smg-4/UPF3 family protein	-			
Glyma.16G002400	shaggy-like protein kinase 41	131448	C	T	Missense variant
Glyma.16G002500	chloroplast heat shock protein 70-2	-			
Glyma.16G002700	-	142145	G	A	Missense variant
		142153	A	G	Missense variant
Glyma.16G003000	DNA-binding bromodomain-containing	-			
Glyma.16G003100	Pseudouridine synthase family protein	-			
Glyma.16G003200	evolutionarily conserved C-terminal region 11	173715	C	G	Missense variant
		175190	C	G	Missense variant
Glyma.16G003300	-	-			
Glyma.16G003600	Protein of unknown function (DUF1682)	198451	C	A	Missense variant
		198520	A	C	Missense variant
		198544	T	C	Missense variant
Glyma.16G003700	Transducin/WD40 repeat-like superfamily	-			
Glyma.16G003900	Protein of unknown function (DUF803)	-			
Glyma.16G004000	-	-			
Glyma.16G004200	-	-			
Glyma.16G004300	TCP family transcription factor	239262	C	G	Missense variant
Glyma.16G004400	Dihydrolipoamide acetyltransferase, long form	251248	A	G	Missense variant
Glyma.16G004800	ATPases;nucleotide binding	270006	C	T	Missense variant
		270015	A	G	Missense variant
		287001	A	G	Missense variant
		287919	T	C	Missense variant
		288509	G	A	Missense variant
Glyma.16G005100	-	349062	G	T	Splice acceptor
		349809	T	G	Missense variant
		349961	G	T	Stop_lost
Glyma.16G005200	Mitochondrial substrate carrier family protein	361425	C	T	Missense variant
Glyma.16G005300	NAD(P)-binding Rossmann-fold superfamily	366194	C	T	Missense variant,
Glyma.16G005400	alfin-like 5	-			
Glyma.16G005600	transcription coactivators	388652	A	T	Missense variant
Glyma.16G005700	CTC-interacting domain 3	-			
Glyma.16G005800	phospholipid:diacylglycerol acyltransferase	431114	T	A	Missense variant
Glyma.16G006000	Kinase-related protein of unknown function	-			
Glyma.16G006100	-	-			

W, the wild parent *N24852*; C, the cultivated parent *NN1138-2*.
